# Emerging Biomarkers in Heart Failure and Cardiac Cachexia

**DOI:** 10.3390/ijms151223878

**Published:** 2014-12-22

**Authors:** Goran Loncar, Daniel Omersa, Natasa Cvetinovic, Aleksandra Arandjelovic, Mitja Lainscak

**Affiliations:** 1 Clinical Hospital Zvezdara, Cardiology Department, Dimitrija Tucovica 161, Belgrade 11000, Serbia; E-Mails: loncar_goran@yahoo.com (G.L.); ncvetinovic@gmail.com (N.C.); drarandjelovic@yahoo.com (A.A.); 2 National Institute of Public Health, Ljubljana 1000, Slovenia; E-Mail: dane.omersa@gmail.com; 3 School of Medicine, University of Belgrade, Belgrade 11000, Serbia; 4 Department of Cardiology, General Hospital Celje, Oblakova 5, Celje 3000, Slovenia; 5 Faculty of Medicine, University of Ljubljana, Ljubljana 1000, Slovenia

**Keywords:** heart failure, cardiac cachexia, emerging biomarkers

## Abstract

Biomarkers are objective tools with an important role for diagnosis, prognosis and therapy optimization in patients with heart failure (HF). To date, natriuretic peptides are closest to optimal biomarker standards for clinical implications in HF. Therefore, the efforts to identify and test new biomarkers in HF are reasonable and justified. Along the natural history of HF, cardiac cachexia may develop, and once at this stage, patient performance and prognosis is particularly poor. For these reasons, numerous biomarkers reflecting hormonal, inflammatory and oxidative stress pathways have been investigated, but only a few convey relevant information. The complex pathophysiology of HF appears far too complex to be embraced by a single biomarker; thus, a combined approach appears reasonable. With these considerations, we have reviewed the recent developments in the field to highlight key candidates with diagnostic, prognostic and therapy optimization properties, either alone or in combination.

## 1. Introduction

**Figure 1:**
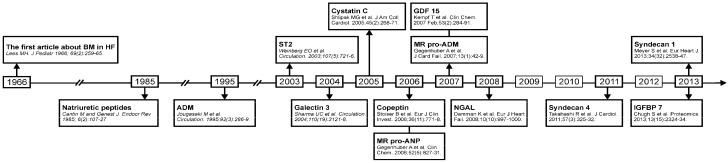
Evolution of the detection of cardiac biomarkers. ADM, adrenomedullin; BM, biomarker; GDF 15, growth-differentiation factor 15; HF, heart failure; IGFBP, insulin-like growth factor binding protein 7; MR-proADM, mid-regional pro-hormone fragment; MR-proANP, mid-regional zone of proANP; NGAL, neutrophil gelatinase associated lipocalin.

**Figure 2:**
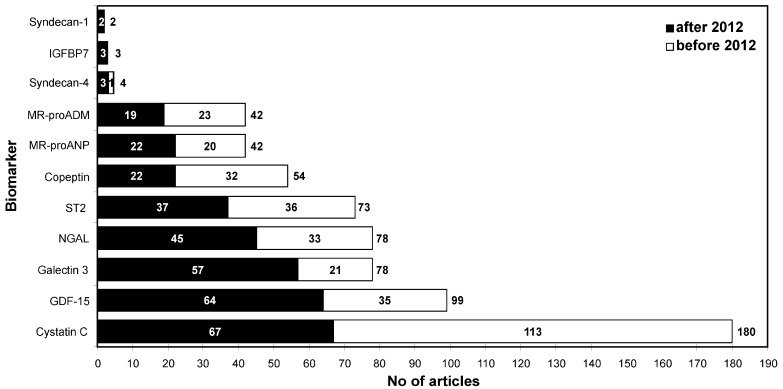
Number of publications with emerging biomarkers of heart failure (HF) before and after 2012 (PubMed database). ADM, adrenomedullin; GDF 15, growth-differentiation factor 15; IGFBP, insulin-like growth factor binding protein 7; MR-proADM, mid-regional pro-hormone fragment; MR-proANP, mid-regional zone of proANP; NGAL, neutrophil gelatinase associated lipocalin.

Heart failure (HF) is a major health problem, because it is common, costly and has a high rate of rehospitalization and high mortality. It is a clinical condition, which is usually defined as a syndrome in which patients have typical symptoms and signs resulting from an abnormality of cardiac structure or function [1]. Biomarkers are objective tools that have an important role in the diagnosis, prognosis and guiding therapy of HF. Since natriuretic peptides (NPs) appeared in the context of HF in 1985 [2], they have been best validated and established as biomarkers of HF [3] (Figure 1). Their diagnostic, prognostic and therapeutic values are strongly confirmed. Although NPs represent the gold standard for biomarkers in HF, they have several important limitations. Factors influencing the clinical interpretation of NPs’ values include advancing age, obesity, renal failure, atrial arrhythmias, cardiotoxic agents, as well as structural heart disease beyond the clinical diagnosis of HF [4]. In 2007, The National Academy of Clinical Biochemistry set comparable goals in a consensus document that states that a biomarker in HF ideally enables clinicians to: “identify possible underlying (and potentially reversible) causes of HF; confirm the presence or absence of the HF syndrome; and estimate the severity of HF and the risk of disease progression” [5]. Nevertheless, in addition to NP, none of the presently available or studied biomarkers meet these standards that have been set for the clinical utilization of cardiac biomarker testing in HF [6]. In light of these facts, the efforts to find and evaluate other biomarkers in HF are reasonable and needed. In the last few years, many new biomarkers have been considered, and this review will discuss emerging biomarkers that are found as promising and the most common in the literature in the recent past (Figure 2).

## 2. Biomarkers in Heart Failure

### 2.1. Mid-Regional Zone of proANP (MR-proANP)

Production of atrial natriuretic peptide (ANP), the first NP to be described, is, like in other NPs, also increased in response to increased atrial wall stretching in HF. While ANP’s major limitation is analytical instability and a short half-life [4,6], the prohormone of ANP (proANP) has a longer half-life, which makes measurement more feasible, and a novel assay, which detects the mid-regional zone of proANP (MR-proANP) [6]. The diagnostic value of MR-proANP was confirmed in the BACH trial, which included 1641 patients with acute dyspnea, where MR-proANP was found to be as useful as B-type natriuretic peptide (BNP) in the diagnosis of acute HF and appeared to improve diagnostic accuracy in the BNP grey zone (levels between 100 and 500 pg/mL) and in patients with obesity [7]. Moreover, in the PRIDE study, MR-proANP was an independent predictor of HF diagnosis in a model that included *N*-terminal of the prohormone brain natriuretic peptide (NT-proBNP) and correctly reclassified both false negatives and false positives and, therefore, confirmed its diagnostic accuracy for HF [8]. Furthermore, the PRIDE study also showed the independent predictive value of MR-proANP for four-year mortality. After these favorable studies, MR-proANP entered into the European Society of Cardiology (ESC) guidelines on HF diagnosis in the acute setting with equal importance as gold standard biomarkers BNP/NT-proBNP [1]. It has been suggested that the combined use of MR-proANP and either BNP or NT-proBNP provides superior diagnostic accuracy than either one alone [4]. Due to the fact that MR-proANP has the same role as BNP in the pathophysiology of HF and similar diagnostic and prognostic value, the hypothesis that it may be used in biomarker-guided therapy is worth testing (Table 1).

**Table 1:** Emerging biomarkers in HF: its evaluation in the diagnosis, prognosis, prediction and making therapy decisions up to date.

Biomarker	Diagnosis		Prognosis			Increased Risk of HF	Making Therapy Decision
	AHF	HFPEF	AHF	CHF	HFPEF		
MR-proANP	+		+				+
ST2	+		+				+
Galectin 3		+	+	+	+		+
MR-proADM	+		+	+		+	+
Copeptin	+		+	+		+	+
GDF15		+		+	+	+	
Cystatin C			+	+	+		+
NGAL			+				+
Procalcitonin							+
Syndecan 1					+		
Syndecan 4				+			
IGFBP 7				+			

AHF, acute heart failure; CHF, chronic heart failure; GDF 15, growth-differentiation factor 15; HF, heart failure; HFPEF, heart failure with preserved ejection fraction; IGFBP, insulin-like growth factor binding protein 7; MR-proADM, mid-regional pro-hormone fragment; MR-proANP, mid-regional zone of proANP; NGAL, neutrophil gelatinase associated lipocalin.

### 2.2. ST2

ST2 is a member of the interleukin (IL)-1 receptor family [9,10], which has an immunomodulatory function as a cell-surface marker of the T helper type 2 lymphocyte [11] and was initially described in the context of cell proliferation, inflammatory states and autoimmune diseases [9]. ST2 includes two forms: a membrane-bound (ST2L) and a soluble ST2 form (sST2) [9]. The functional ligand for sST2 and ST2L is IL-33, which stimulates antihypertrophic, antifibrotic and antiapoptotic effects [12]. ST2L mediates the beneficial effects of IL-33, which results in resistance to apoptosis and reduction in fibrosis [12]. In contrast, sST2 is implicated in the attenuation of Th2 inflammatory responses, and it is thought to function as a decoy receptor, neutralizing the benefits of IL-33 [10].

In 2002, it was noted that the transcript for ST2 was markedly upregulated in mechanically-stimulated cardiomyocytes [13]. Both the trans-membrane and soluble forms of ST2 were induced, with the sST2 displaying a more robust expression [13]. The next year, the results of the PRAISE-2 HF trial (The New York Heart Association (NYHA) Functional Classification—NYHA class III-IV; end point mortality or transplantation) indicated that the change in ST2 remained significant as a predictor of mortality or transplantation independent of BNP and proANP [9]. These results identified the serum sST2 as a novel biomarker for neurohormonal activation in patients with HF [9]. Since then, many studies have confirmed the prognostic value of sST2 in HF patients (Table 1) [14,15,16,17].

sST2 has only a minor value for diagnostic purposes [14,16,18]. Compared to other biomarkers, such as NP, the advantages of sST2 include its concentration not being affected by age, renal function or body mass index [18]. Although sST2 seems to be more specific, it is less sensitive than BNP for acute HF (Table 2) [19]. sST2 has the potential to be a marker for therapy guides, but it requires further evaluation [20].

**Table 2:** Optimal cutoff values of biomarkers with sensitivity and specificity for the diagnosis of HF.

Biomarker	Cutoff Value	Sensitivity (%)	Specificity (%)	AUC
MR-proANP [7]	120 pmol/L	90–97	59.9–68	0.88
ST2 [19]	34.3 U/mL	73.5	79.6	0.75
Galectin 3 [21]	17.8 ng/mL	94.3	65.1	0.72
GDF 15 [22]	1306 ng/mL	71.2	68.8	0.76

AUC, area under the curve; GDF 15, growth-differentiation factor 15; MR-proADM, mid-regional pro-hormone fragment; MR-proANP, mid-regional zone of proANP.

### 2.3. Galectin 3

Galectin-3 is a member of the lectin family, which is found in a wide variety of cells and tissue surfaces [4]. It is thought to represent a link between inflammation and fibrosis [22]. Galectin-3 is secreted by activated macrophages and is especially localized at sites of fibrosis and fibroblasts [23]. Recombinant galectin-3 *in vitro* stimulates proliferation and collagen production of cardiac fibroblasts [24]. Studies have shown that galectin-3 genetic knockout mouse models are resistant to left ventricular pressure and volume overload, possessing a slower progression to LV dysfunction or HF [25]. Moreover, the increased myocardial expression of galectin-3 has been found in rats, which later rapidly progressed to HF [24].

Because the levels of galectin-3 are increased in patients with acute HF, galectin-3 has been proposed as a novel biomarker for the diagnosis (Table 2) and prognosis of acute HF. It may also help to establish the diagnosis of HF with the preserved ejection fraction (HFPEF) in patients presenting exercise intolerance [26] and is especially predictive for mortality in HFPEF patients (Table 1) [27]. Although NT-proBNP outperforms galectin-3 for the diagnosis of acute HF, galectin-3 is superior to NT-proBNP for a 60-day mortality prediction [28]. The combination of an elevated galectin-3 level and NT-proBNP has been shown to be a better predictor of mortality than any of the two markers alone [28]. Furthermore, its long-term prognostic value was confirmed in the DEAL-HF study (NYHA class III) [29]. In addition, elevated galectin-3 was found to be an independent predictor of adverse HF outcomes even in patients who had mildly symptomatic HF (NYHA class I/II) [30].

The data suggest that galectin-3 may be used to drive therapeutical strategy [31,32]. Elevated circulating levels of galectin-3 appear to modify the response to certain pharmacological therapies, such as statin and angiotensin II receptor antagonist therapy [31]. A multi-center, randomized study, the MADIT-CRT (CRT with ICD *vs.* ICD only), suggests that galectin-3 might identify the highest risk HF patients who may derive the greatest absolute benefit from CRT-D therapy [30].

### 2.4. Mid-Regional Pro-Hormone Fragment of Adrenomedullin (MR-proADM)

Adrenomedullin (ADM) is a neurohormone with natriuretic, vasodilatory and hypotensive effects mediated by cyclic adenosine monophosphate (cAMP), nitric oxide and renal prostaglandin systems [33]. ADM is up-regulated in HF as an endogenous compensatory mechanism for hemodynamic abnormalities [4]. It has a short half-life and circulates in a bound form that is difficult to measure. Its mid-regional pro-hormone fragment (MR-proADM), whose levels correspond to ADM, was identified as stable in plasma and more feasible to measure [34]. In older patients, the addition of MR-proADM to NT-proBNP improves the diagnostic accuracy of acute HF [35]. In a healthy general population (sample of the FINRISK 1997 cohort), MR-proADM significantly predicted HF even beyond NT-proBNP with improved risk reclassification for HF [36]. The prognostic role of MR-proADM is confirmed both in patients with acute and chronic HF, particularly for a short-term prognosis (Table 1). In the BACH study, it powerfully predicted death at 90 days and one year in patients with acute HF [7]. A potential role of MR-proADM in biomarker-guided therapy has not been evaluated yet, and a suggestion that it may be useful for monitoring acute responses to therapy in HF deserves to be tested [37].

### 2.5. Copeptin

Arginine vasopressin (AVP) is an antidiuretic and vasoconstrictive hormone, released from the hypothalamus and upregulated in HF [6,38]. Due to AVP’s short half-life and instability *in vitro* [6], copeptin (CTproAVP), the *C*-terminal portion of provasopressin with long stability and levels directly proportional to AVP levels, can be used as a surrogate biomarker of AVP secretion [6]. Increased copeptin levels have been described in several studies as a strong predictor of mortality in patients with chronic or acute HF [39]. Furthermore, copeptin is able to predict prognosis independently from troponin or NT-proBNP [6] and is also associated with an increased risk of HF [40]. A recently published study, which included patients with dyspnea, indicated the diagnostic potential of copeptin for acute heart failure [41]. Although the median level of copeptin in patients with acute HF was significantly higher than in patients without acute HF, its level has significant predictive value for 90-day death and rehospitalization [41]. Copeptin has a potential role in the biomarker-guided therapy of HF. It appears to have the potential to monitor acute responses to therapy [39,42].

### 2.6. Growth-Differentiation Factor 15 (GDF 15)

Growth-differentiation factor 15 (GDF 15) is a member of the transforming growth factor β (TGF-β) cytokine superfamily, which has emerged as a promising cardiovascular biomarker. GDF 15 is weakly produced under baseline conditions in most tissues [43]. However, in response to pathologic or environmental stress, GDF15 production may increase. GDF 15 production increases markedly in the heart in mouse models of myocardial infarction and HF [43]. Serum GDF 15 levels also correlate positively with LV mass and the reduced LV ejection fraction in elderly individuals [44] and correlate with the severity of myocardial fibrosis found in patients with end-stage non-ischemic dilated cardiomyopathy [45].

Regarding its diagnostic value, GDF 15 levels are higher in patients with HF than in healthy individuals [43]. In addition, GDF 15 is closely correlated with the severity of HF [43,46]. In morbidly obese individuals, GDF 15 levels seem to better correlate with diastolic dysfunction than NT-proBNP levels [47]. A study that had included HF patients of the NYHA class III showed GDF 15 to be one of the most predictive markers for long-term mortality, even stronger than NT-proBNP [48]. The serum GDF 15 may be a useful predictor of disease severity and poor prognosis in patients with HFPEF [49]. Moreover, increased levels of GDF 15 are associated with an increased risk of developing HF in apparently healthy individuals [50].

Although previous studies have confirmed the prognostic value of GDF 15, the evidence for diagnostic and therapeutic utility is poor. In a subgroup of chronic heart failure (CHF) patients from the ongoing DIAST-CHF observational trial, it has been shown that diagnostic precision of GDF 15 for HFPEF is at least as good as that of NT-proBNP and that combining both markers improves diagnostic accuracy [51].

### 2.7. Biomarkers of Extracardiac Involvement

#### 2.7.1. Renal Impairment in HF

Patients with HF have a significant decline of their renal function. Changes in renal function are an important diagnostic and prognostic indicator in HF patients. Cystatin-C and NGAL are confirmed as biomarkers for acute kidney injury (AKI) [52]. Their diagnostic and prognostic values are well validated in the setting of HF. Furthermore, they may show similar promise for informing therapeutic decision making in patients with HF. In theory, the use of an AKI marker could supplement therapeutic decision making in the context of potentially nephrotoxic and/or nephroprotective therapies and strategies [6]. In general, using biomarkers for individual therapy tailoring could improve current treatment strategies, and some new biomarkers deserve to be better evaluated in this context in the future (Table 3).

**Table 3:** Emerging biomarkers in the context of therapy tailoring in HF patients.

Determining Therapy Approach	Monitoring Responses to Therapy	Therapy Guiding Potential
Galectin 3	MR-proADM	MR-proANP
Cystatin-C	Copeptin	ST2
NGAL		

MR-proADM, mid-regional pro-hormone fragment; MR-proANP, mid-regional zone of proANP; NGAL, neutrophil gelatinase associated lipocalin.

Cystatin-C is a serine protease inhibitor that is released from all functioning cells. Clearance of cystatin-C depends entirely on glomerular filtration, making it a prototype marker of renal function [1,53]. Although cystatin-C is slightly superior to estimated glomerular filtration rate (eGFR) in detecting renal impairment, increased concentrations are not only indicative of renal dysfunction, but may also be influenced by inflammation, as well as the presence and severity of underlying heart disease [54]. It has been shown that cystatin-C positively correlates with NT-proBNP in patients with acute HF and AKI, and it represents an independent predictor of one-year mortality for acute HF patients [55]. Moreover, it has been shown that cystatin-C is an independent risk factor in the prognosis of patients with HF [56] and has a significant prognostic value in patients with HF and an ejection fraction (EF) of left ventricle >40% and in stable HF patients who had a lower EF <35% [57]. Although the diagnostic value of cystatin-C for AKI is confirmed [58], as well as the prognostic value for HF patients, its potential role in the diagnosis of HF has also been under consideration. A hypothesis, which has appeared recently, assumes the differences in serum levels of urea and cystatin-C as being useful in the diagnosis of HF [59].

NGAL (neutrophil gelatinase associated lipocalin) is a protein normally expressed in neutrophils and in low levels in the kidney, prostate and epithelia of the respiratory and alimentary tracts [60]. Its expression is highly upregulated at an early stage of kidney injury, and NGAL can be rapidly detected in the circulation or in urine [61]. Many studies have shown a role of NGAL as an early diagnostic marker for AKI. Although NGAL has been compared as a biomarker of kidney injury to cardiac troponin as a marker of heart injury, it is not specific for the kidney [62]. It has a role in the diagnosis, management and follow-up of patients with cardiorenal syndromes [63]. It appeared in an HF context in 2008 (Figure 1); since then, it has become one of most evaluated biomarkers (Figure 2). NGAL is increased in HF patients compared to healthy controls [64]. Its levels on admission are predictive for post-discharge outcomes in patients with acute HF [65]. Furthermore, plasma NGAL predicts mortality in HF patients, both in patients with and without chronic kidney disease, and is a stronger predictor for mortality than the established renal function indices of eGFR and cystatin-C [66]. The GALLANT trial assessed the utility of plasma NGAL in acute HF [67]. Plasma NGAL is a measure of kidney injury at the time of discharge and a strong prognostic indicator for HF rehospitalization and all-cause mortality in the 30 days following admission for acute HF [67]. The presence of elevated admission serum NGAL levels is associated with heightened risk of the subsequent development of worsening renal failure in patients admitted with acute decompensated HF [68]. This early notification of renal function aggravation may be helpful in treatment optimization of these patients, especially with respect to the timing of the dosage change of diuretics and vasodilators.

#### 2.7.2. Procalcitonin

In 1999, congestive HF patients were described to have slightly raised mean procalcitonin (PCT) concentrations compared to healthy controls [69]. It is known that PCT, as a high specific marker of an infectious etiology, such as pneumonia or chronic obstructive pulmonary disease exacerbation, may be helpful for emergency department physicians in the differential diagnosis and risk stratification of patients presenting acute dyspnea [70]. Moreover, it has been suggested that complicated HF elevates the PCT levels in patients with bacterial infections [71]. Certainly one of most important roles of PCT in an HF context is its potential for guiding the diagnosis and treatment of HF patients with possible acute respiratory infections (Table 1) [72].

### 2.8. The Newest Biomarkers of HF

Syndecan-1 and syndecan-4 are members of the proteoglycan family involved in cell-matrix interactions and heart remodeling, which have appeared as promising HF biomarkers within the last three years (Figure 1 and Figure 2). Recently, it has been shown that syndecan-1 is associated with clinical outcomes in HFPEF patients, but not in HFREF patients [73]. In the COACH study, syndecan-1 has had a sex-dependent prognostic value in HF patients. Women were found to have lower levels of syndecan-1, which were associated with poor outcomes [74]. Moreover, levels of syndecan-4 have been shown to be significantly increased in CHF patients. They were closely correlated with NYHA class and left ventricle (LV) function parameters. Thus, syndecan-4 levels may have an important value in the detection and surveillance of HF status [75]. Insulin-like growth factor binding protein 7 (IGFBP 7) is a recently discovered urinary biomarker for AKI [76], which has also been presented as an excellent candidate plasma biomarker of HF [77,78]. MicroRNAs, non-coding RNA molecules that are post-transcriptional modulators of gene expression, have an emerging role in cardiovascular diseases [79,80] and a potential for the diagnosis and prognosis of HF patients [81]. Furthermore, modulation of microRNA expression and activity may be a therapeutic target in these patients [82].

## 3. Biomarkers of Cardiac Cachexia in Heart Failure

A consensus statement from 2008 proposed to clinically define cachexia as a non-edematous weight loss exceeding 5% within the previous 3–12 months in combination with symptoms characteristic of cachexia (e.g., fatigue or depression), loss of lean body mass and biochemical abnormalities (e.g., anemia or inflammation) associated with chronic diseases [83]. In CHF patients, the prevalence of cardiac cachexia (CC) was 5%–15% [84].

CC is as an important comorbidity of HF patients and an independent factor for survival reduction [85]. It is associated with poor prognosis, independently of functional disease severity, age, measures of exercise capacity and LV EF [86]. Complex imbalance of catabolic and anabolic body systems is likely to be responsible for the development of the wasting process [86]. This imbalance is caused by a series of immunological, metabolic and neurohormonal processes, most of which are activated early in the development of CHF [87]. Recent research has led to recognizing the complexity of the metabolic aspects of HF pathophysiology. Furthermore, in patients with stable HF, the blood flow in the intestinal arteries is reduced and relates to CC [88]. Muscle wasting may manifest in the form of loss of muscle mass and function, termed sarcopenia in healthy aging. Triggers for muscle wasting are more numerous and include a general activation of the sympathetic nervous system, pro-inflammatory cytokines, angiotensin-II, glucocorticoids and members of the TGF-β family [84].

Currently, wasting assessment is limited only to quantification of muscle mass based on imaging and functional tests to quantify muscle function. Unfortunately, all are cost-intensive and only available at medical centers equipped to do so; what is more, such tests only allow for wasting detection, but not for patients at risk of developing muscle atrophy [89,90]. Therefore, the identification of reliable biomarkers that can be used in a cost-effective manner and could guide diagnosis and therapy in routine clinical practice and clinical trials is an important part of further investigations (Table 4). Some biomarkers mainly associated with hormonal, inflammatory and oxidative stress changes have been proposed for the assessment of cardiac cachexia; however, they present only a potential prognostic value.

**Table 4:** Candidates for biomarkers of cardiac cachexia.

Biomarkers of Cardiac Cachexia
Ghrelin
Adiponectin
*C*-terminal agrin fragment (CAF)
Growth differentiation factor 15 (GDF 15)
Atrial natriuretic peptide (ANP)
*N*-terminal propeptide of type III procollagen (P3NP)
Type VI collagen *N*-terminal globular domain epitope
Myostatin

Ghrelin: The resistance of HF patients to the effects of appetite-stimulating peptide ghrelin may be one of the contributing factors in the development of CC [91]. Patients with CHF and CC have higher plasma ghrelin levels than in those without CC and healthy subjects, which may be suggestive of a compensatory mechanism under the conditions of anabolic/catabolic imbalance, countering further energy deficit and defending against starvation [92]. The results of preliminary studies support the clinical potential of ghrelin, ghrelin gene-derived peptides and ghrelin receptor agonists, suggesting that larger clinical trials are demanded [93].

Adipokines: Plasma levels of the adipokines, leptin and adiponectin, may have a role in the detection of muscle, bone and fat wasting processes [91,94]. Cachexia in HF is associated with an increase in adiponectin concentration. It may suggest that adiponectin plays a role in the pathogenesis of CC [95,96]. Studies have confirmed that the direct correlation between serum leptin levels and severity of CHF is present in CHF, including CC, as patients with CC have plasma leptin concentrations lower than those without it [93].

CAF: *C*-terminal agrin fragment (CAF) from the peptide agrin has been proposed as a biomarker of muscle wasting. CAF is produced by the enzyme neurotrypsin and is a synaptically located key player during the initial formation and maintenance of neuromuscular junctions [97]. Furthermore, it has been proposed as a novel diagnostic marker for muscle wasting in CHF patients, which may be useful in identifying patients with CC, prompting further investigations in these patients [98].

GDF 15: Some studies have shown that GDF 15, which has also been proposed as a novel biomarker for HF, plays an important role in the pathways of muscle wasting and cachexia. Recent findings suggest that GDF 15 induces weight, muscle and fat loss, as well as that it decreases activity in mice and may be a promising target for therapeutic interventions in the field of cachexia and muscle wasting [97,99].

ANP: This is another biomarker of HF, which has been proposed as biomarker of CC [100].

P3NP: This collagen fragment *N*-terminal propeptide of type III procollagen (P3NP) is a measure of skeletal muscle status [101] and a biomarker candidate for muscle anabolism. It is released into circulation during collagen synthesis in soft lean tissue, and its levels have been described to be associated with subsequent changes in the lean mass of elderly patients [89,102]. The type VI collagen *N*-terminal globular domain epitope, which is a degradation fragment of collagen VI, has been identified as a novel biomarker of muscle mass or change in muscle mass in young men [103,104].

Myostatin: Myostatin, also known as growth and differentiation factor 8, is well characterized as a negative regulator of muscle growth and has been implicated in several forms of muscle wasting, including severe cachexia [97]. Although it seems a natural candidate for an atrophy biomarker [105], as it directly mediates catabolic signaling [106], the data of a recent study in CC could not confirm the role of circulating myostatin as a biomarker for muscle wasting in humans [89].

Creatinine: Serum creatinine under steady-state conditions has been suggested to serve as a reliable muscle mass biomarker, if appropriate adjustment for kidney function and dietary meat intake is undertaken [89]. Recently, a new method has been validated in cross-sectional studies to determine total body creatine pool size and skeletal muscle mass based on a heavy water-labeled tracer, creatinine-(methyl-d(3)), *i.e.*, d_3_-creatine, dilution from an oral dose and detection of urinary creatinine enrichment by isotope ratio mass spectrometry. It has been shown that the d_3_-creatine dilution method may be used for longitudinal assessment of changes in skeletal muscle mass [107].

Although a number of plasma assessable biomarkers for CC have been proposed, there is need for further investigations. An ideal biomarker of CC has to be well validated, sensitive, specific, low cost and should be able to distinguish between cachexia and sarcopenia (loss of muscle mass due to aging), in CHF patients, due to different prognostic value and treatment implications. The complex biochemical network associated with CHF and CC pathophysiology suggests that a single biomarker cannot reflect all of the features of the disease. Based on these limitations, future studies should be focused on the use of a combination of multiple biomarkers in order to establish an ideal panel that better reflects all of the features of the syndrome [100].

## 4. Conclusions and Future Research

Many promising novel biomarkers of HF have been proposed; yet, the NPs are still the best validated and, despite some limitations, represent the current gold standard in clinical practice. The field of emerging HF biomarkers is rich and reflects different mechanism of HF development and progression. Although none of the presently available biomarkers, excluding NPs, meets all standards that have been set by The National Academy of Clinical Biochemistry for the clinical utilization of biomarker testing in HF, several biomarkers have significant potential. The complexity of the biochemical network at the basis of HF pathophysiology clearly suggests that it is unrealistic that a single marker is able to reflect all of the features of this syndrome, whereas the combined use of more parameters would certainly give more comprehensive insight into an individual patient [108,109]. According to these facts, a multiple biomarker strategy should become feasible to improve diagnostic and prognostic accuracy in HF patients, as well as allow individualized therapy. The choice of biomarker combination is essential to the performance of a multimarker strategy, and future research should be focused on finding the most appropriate combination of biomarkers. Certainly, some emerging biomarkers may play an important role in the multimarker approach.
